# Ribonuclease inhibitor 1 emerges as a potential biomarker and modulates inflammation and iron homeostasis in sepsis

**DOI:** 10.1038/s41598-024-65778-8

**Published:** 2024-06-28

**Authors:** Carolina Neu, Christian Beckers, Nadine Frank, Katharina Thomas, Matthias Bartneck, Tim-Philipp Simon, Jana Mossanen, Kimmo Peters, Tobias Singendonk, Lukas Martin, Gernot Marx, Sandra Kraemer, Elisabeth Zechendorf

**Affiliations:** 1 Department of Intensive and Intermediate Care, University Hospital RWTH Aachen, Pauwelsstraße 30, 52074 Aachen, Germany; 2 Department of Medicine III, University Hospital RWTH Aachen, 52074 Aachen, Germany

**Keywords:** Sepsis, Ribonuclease inhibitor 1, Inflammation, Iron homeostasis, Prognostic markers, Sepsis

## Abstract

Sepsis, marked by organ dysfunction, necessitates reliable biomarkers. Ribonuclease inhibitor 1 (RNH1), a ribonuclease (RNase) inhibitor, emerged as a potential biomarker for acute kidney injury and mortality in thoracoabdominal aortic aneurysm patients. Our study investigates RNH1 dynamics in sepsis, its links to mortality and organ dysfunction, and the interplay with RNase 1 and RNase 5. Furthermore, we explore RNH1 as a therapeutic target in sepsis-related processes like inflammation, non-canonical inflammasome activation, and iron homeostasis. We showed that RNH1 levels are significantly higher in deceased patients compared to sepsis survivors and correlate with creatine kinase, aspartate and alanine transaminase, bilirubin, serum creatinine and RNase 5, but not RNase 1. RNH1 mitigated LPS-induced TNFα and RNase 5 secretion, and relative mRNA expression of ferroptosis-associated genes HMOX1, FTH1 and HAMP in PBMCs. Monocytes were identified as the predominant type of LPS-positive PBMCs. Exogenous RNH1 attenuated LPS-induced CASP5 expression, while increasing IL-1β secretion in PBMCs and THP-1 macrophages. As RNH1 has contradictory effects on inflammation and non-canonical inflammasome activation, its use as a therapeutic agent is limited. However, RNH1 levels may play a central role in iron homeostasis during sepsis, supporting our clinical observations. Hence, RNH1 shows promise as biomarkers for renal and hepatic dysfunction and hepatocyte injury, and may be useful in predicting the outcome of septic patients.

## Introduction

In accordance with the most recent definition, sepsis is a life-threatening organ dysfunction caused by a dysregulated host response to infection (sepsis-3)^1^. With one in five deaths worldwide attributed to sepsis in 2017, it is still a leading cause of death in critically ill patients^2^. Multi-organ failure is caused by circulatory dysfunction inducing an imbalance between blood flow and the metabolic needs of the tissue and is directly linked to mortality^3^.

As the most common trigger of sepsis, the bacterial endotoxin LPS causes an excessive inflammatory response through both intracellular and extracellular pathways^4^. LPS, a pathogen-associated molecular pattern, is recognized by the extracellular pattern recognition receptor toll-like receptor (TLR4)^5^. Activation of TLR4 triggers the release of tumor necrosis factor α (TNFα) and other inflammatory cytokines via the translocation and activation of nuclear factor κB^6,7^. Intracellular LPS signaling occurs through recognition by guanylate-binding proteins (GBPs)^8^. LPS binding triggers caspase-4/-5 (CASP4/5) oligomerization and activation, cleaving the pore-forming protein gasdermin D (GSDMD). This induces pyroptosis and activates the non-canonical inflammasome nucleotide-binding oligomerization domain leucine-rich repeat-containing protein receptor 3 (NLRP3)^9,10^.

Cytokines released in sepsis can impact iron homeostasis, linking to ferroptosis, an iron-dependent regulated cell death^11,12^. Iron is a trace element essential for various physiological functions, such as the metabolism of glucose, lipids, and amino acids, as well as DNA synthesis^13^. It is predominantly present in the form of heme, which is degraded into free iron, carbon monoxide, and bilirubin by heme oxygenase-1 (HMOX1). However, free iron is also implicated in bacterial pathogenicity^14–16^. Ferritin and hepcidin (HAMP) are pivotal regulators of iron metabolism, with ferritin storing iron and HAMP inhibiting its export through ferroportin^17,18^. During sepsis, both ferritin and HAMP are upregulated, restricting iron availability to pathogens as a host defense mechanism^19,20^.

Similarly, ribonuclease (RNase) 1, a ubiquitously expressed host defense peptide, is elevated during sepsis^21^. RNase 1 modulates the inflammatory response to circulating extracellular RNA (eRNA), a danger-associated molecular pattern released from necrotic cells, through catalytic degradation. However, RNase 1 itself is regulated by ribonuclease inhibitor 1 (RNH1)^22,23^. In human tissues, RNH1 is ubiquitously expressed and primarily acts by inhibiting RNases by forming RNH1–RNase complexes with femtomolar affinity^24^. Biologically, RNH1 participates in processes such as angiogenin (RNase 5)-regulated neovascularization, monitoring of the cellular oxidative state, and cancer growth and metastasis^25–27^. In clinical observations of patients undergoing thoracoabdominal aortic aneurysm (TAAA) repair, we previously identified RNH1 as a potential biomarker for acute kidney injury (AKI) and in-hospital mortality^28^. A common complication observed in up to 60% of septic patients is the concurrent development of AKI^29,30^. Patients with sepsis-related AKI display a higher mortality rate (70%) compared to septic patients without AKI (20%)^31^. RNH1 levels are elevated in septic patients compared to healthy subjects, as we previously demonstrated^23^. However, nothing is yet known about the role of RNH1 as a potential biomarker or therapeutic target in sepsis.

Therefore, we aimed to investigate (a) whether the plasma dynamics of RNH1 can serve as a biomarker for worsened outcomes in mortality, renal, and hepatic organ dysfunction in septic patients; (b) the plasma dynamics of RNase 1, RNase 1 activity, and RNase 5 and their direct relationships with RNH1 during sepsis; and (c) the effect of RNH1 as a treatment on LPS-induced inflammation, non-canonical inflammasome activation, and iron homeostasis ex vivo and in vitro.

## Results

### Patients’ characteristics

The study population included 32 patients with a sepsis-3 diagnosis including 21 patients with septic shock. The average age was 63.5 years, and 65.63% were male. Comorbidities were recorded in 87.50% of the patients. For example, 43.75% of the patients had diabetes. On average, patients were hospitalized for 34 days, including 16 days in the intensive care unit (ICU). Mortality in the ICU was 25.00%, and 71.88% of patients suffered from AKI. Eight healthy volunteers of the same age and sex served as a control group. Further details of the patients’ characteristics are presented in Supplemental Table 1.

### RNH1 levels and their correlation with ICU mortality, creatine kinase, AST, ALT, bilirubin and serum creatinine in septic patients

**Figure 1:**
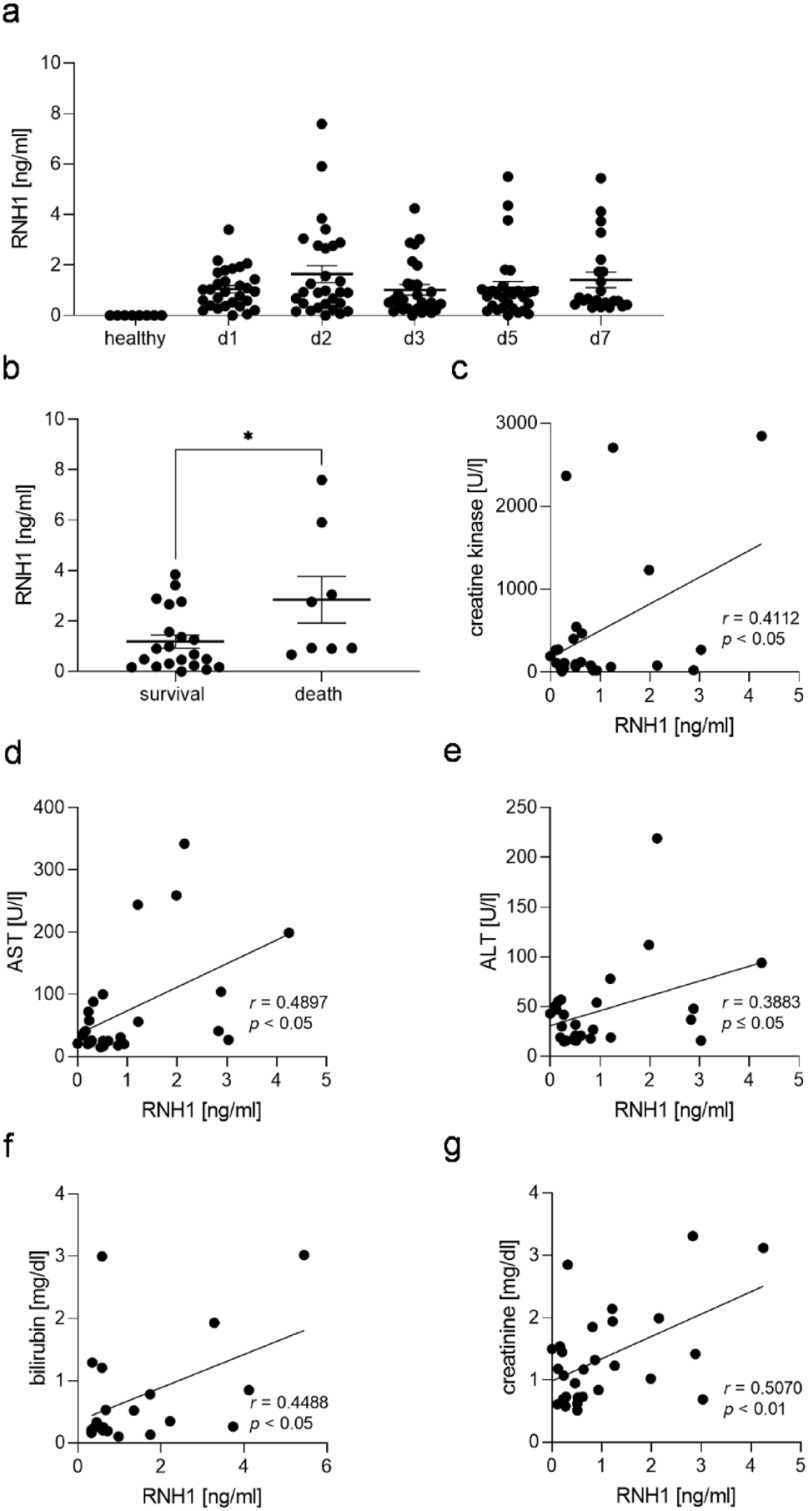
RNH1 levels in septic patients and their correlation with ICU mortality, creatine kinase, AST, ALT, bilirubin and creatinine levels. Presented are (**A**) RNH1 levels on days 1–3, 5, and 7 after sepsis diagnosis, quantified using ELISA; (**B**) the correlation of RNH1 levels on day 2 with ICU mortality, and the correlations of RNH1 levels on day 3 with (**C**) creatine kinase levels on day 3, (**D**) AST, and (**E**) ALT levels on day 5; (**F**) the correlation of RNH1 levels on day 7 with bilirubin levels on day 7, and (**G**) the correlation of RNH1 levels on day 3 with serum creatinine levels on day 3 in septic patients. Two-way ANOVA followed by Tukey’s test, unpaired t-test (two-tailed), or simple linear regression was used for statistical analysis. *p < 0.05; RNH1, ribonuclease inhibitor 1; ICU, intensive care unit; AST, aspartate transaminase; ALT, alanine transaminase.

Herein, we initially analyzed the RNH1 dynamics in septic patients by measuring concentrations on days 1 to 3, 5, and 7 after diagnosis, along with age- and sex-matched healthy volunteers. Compared to healthy volunteers in whom RNH1 was not detectable, we measured increased RNH1 levels in the plasma of septic patients. Interestingly, no significant alterations were observed over time. However, the highest RNH1 levels were measured on day 2 (Fig. 1a). Patients who died during the ICU stay (25%) showed significantly higher RNH1 levels on day 2 compared to survivors (*p* = 0.0060; Fig. 1b). The maximum Sequential Organ Failure Assessment (SOFA) score of 10.47 on day 3 was significantly elevated compared to day 7 (*p* = 0.0112; Supplemental Fig. 1).

Since sepsis is strongly associated with multi-organ failure, we investigated the role of RNH1 in several organ functions. In fact, we observed a significant correlation between RNH1 levels and creatine kinase levels (*p* = 0.0369, r = 0.4112; Fig. 1c) on day 3. Septic patients also showed significantly correlations between RNH1 levels on day 3 and aspartate and alanine transaminase (AST and ALT) levels (*p* = 0.001, r = 0.4897 and *p* = 0.05, r = 0.3883, respectively) on day 5 (Fig. 1d,e). On day 7 after sepsis diagnosis, we observed a correlation between RNH1 levels and bilirubin levels on day 7 (*p* = 0.0361, r = 0.4488; Fig. 1f). Furthermore, we determined a significant correlation between RNH1 levels and serum creatinine levels on day 3 (*p* = 0.0059, r = 0.5070; Fig. 1g). These results indicate that increased RNH1 levels are associated with poorer outcomes and worsened renal and hepatic function in septic patients.

### Dynamics of RNase 1 levels, RNase 1 activity, and RNase 5 levels and their correlation with RNH1 levels in septic patients

**Figure 2:**
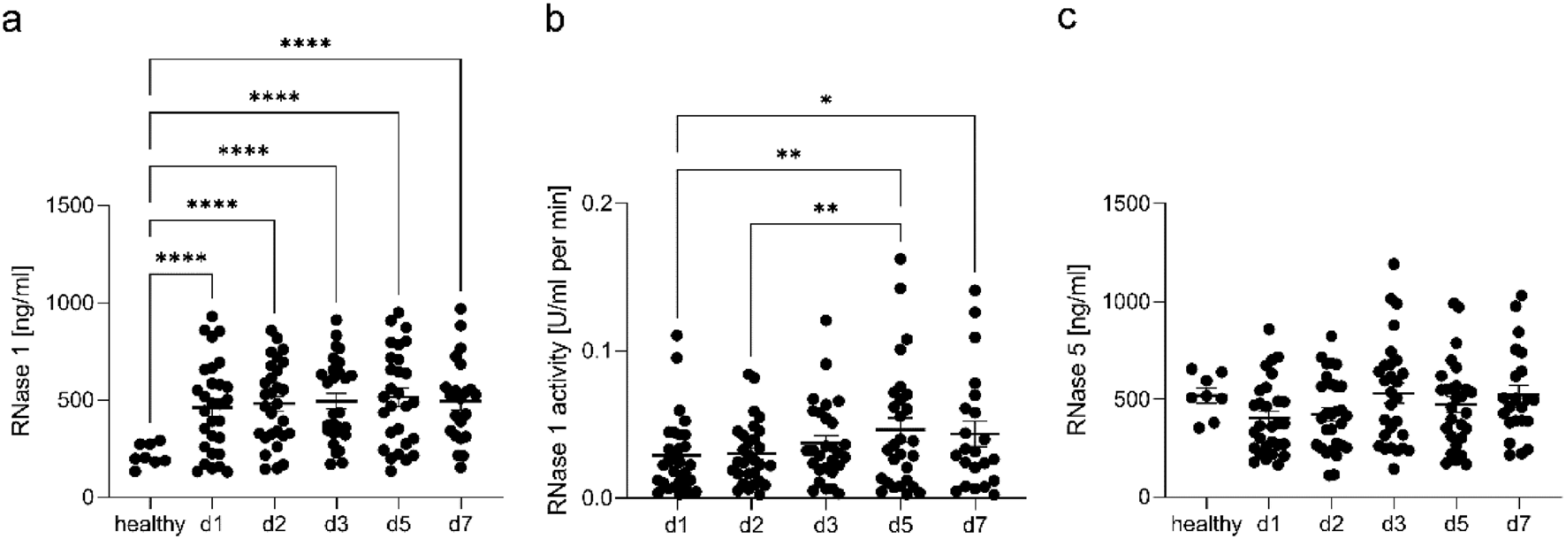
RNase 1 levels, RNase 1 activity and RNase 5 levels in septic patients. Presented are (**A**) RNase 1 levels quantified using ELISA, (**B**) RNase 1 activity measured using an RNase activity assay, and (**C**) RNase 5 levels quantified using ELISA on days 1–3, 5, and 7 after sepsis diagnosis. Two-way ANOVA followed by Tukey’s test was used for statistical analysis. *p < 0.05; **p < 0.01; ****p < 0.0001; RNase 1, ribonuclease 1; RNase 5, ribonuclease 5.

As RNH1 primarily inhibits RNase activity, we next examined its relationship with RNase 1 activity, as well as RNase 1, and RNase 5 levels. Starting from sepsis diagnosis, we measured significantly increased RNase 1 concentrations at all time points compared to healthy volunteers (all *p* < 0.0001; Fig. 2a). While no significant differences in the RNase 1 levels of septic patients were observed between the time points measured (Fig. 2a), we identified significant changes in RNase 1 activity. Specifically, RNase 1 activity increased on day 5 compared to days 1 and 2 (*p* = 0.0023 and *p* = 0.0066) and on day 7 compared to day 1 (*p* = 0.0334; Fig. 2b). Regarding RNase 5 dynamics, no significant alterations were observed between the measured time points or compared to healthy volunteers (Fig. 2c).

Additionally, we explored the correlation between RNase 1 level and RNase 1 activity. Throughout all measured time points, RNase 1 activity significantly correlated with RNase 1 concentrations. Interestingly, no correlations between RNH1 and RNase 1 concentrations or between RNH1 levels and RNase 1 activity were demonstrated at any time point. However, we observed significant correlations of RNH1 and RNase 5 concentrations on days 3 and 7 (Supplemental Table 2). These results suggest that RNH1 concentrations may be directly connected to RNase 5 but not RNase 1 levels during sepsis.

### The effect of exogenous RNH1 on LPS-induced inflammation and RNase 5 levels in PBMCs

**Figure 3:**
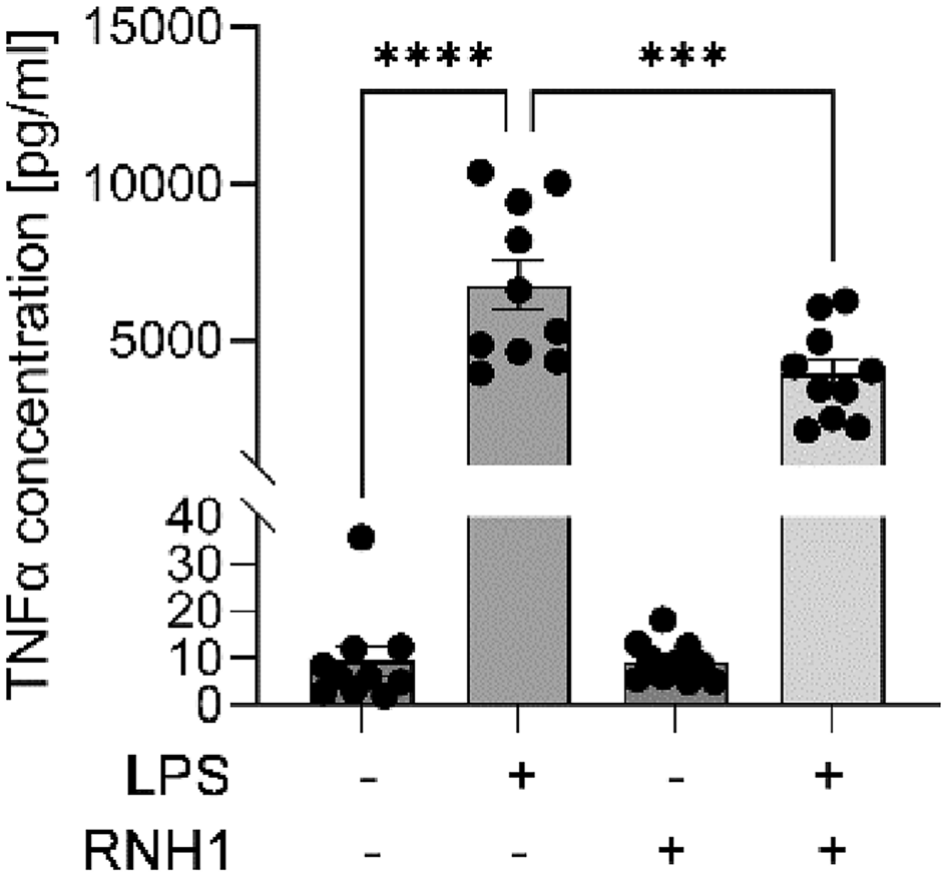
RNH1 attenuates LPS-induced TNFα secretion in PBMCs. Isolated PBMCs exposed to 50 ng/ml LPS in the presence or absence of 640 U/ml RNH1 for 4 h compared to unstimulated cells were analyzed regarding TNFα protein concentrations quantified using ELISA. Data are presented as column bar graphs of the mean ± SEM (n = 10). Repeated-measures one-way ANOVA followed by Tukey’s test was used for multiple comparisons. ***p < 0.001; ****p < 0.0001; RNH1, ribonuclease inhibitor 1; LPS, lipopolysaccharide; TNFα, tumor necrosis factor α; PBMCs, peripheral blood mononuclear cells.

To further investigate the as yet unknown role of RNH1 in sepsis, we analyzed its effect as a stimulus on the inflammatory response to LPS *ex vivo*. We detected significantly elevated TNFα secretion of PBMCs stimulated with LPS, compared to unstimulated cells (*p* < 0.001). The exposure of PBMCs to LPS + RNH1 resulted in significantly decreased TNFα concentrations compared to LPS-stimulated cells (*p* = 0.0008, Fig. 3), indicating an anti-inflammatory effect of RNH1.

### Enrichment analysis of RNH1-regulated differentially expressed genes in LPS-treated PBMCs

**Figure 4:**
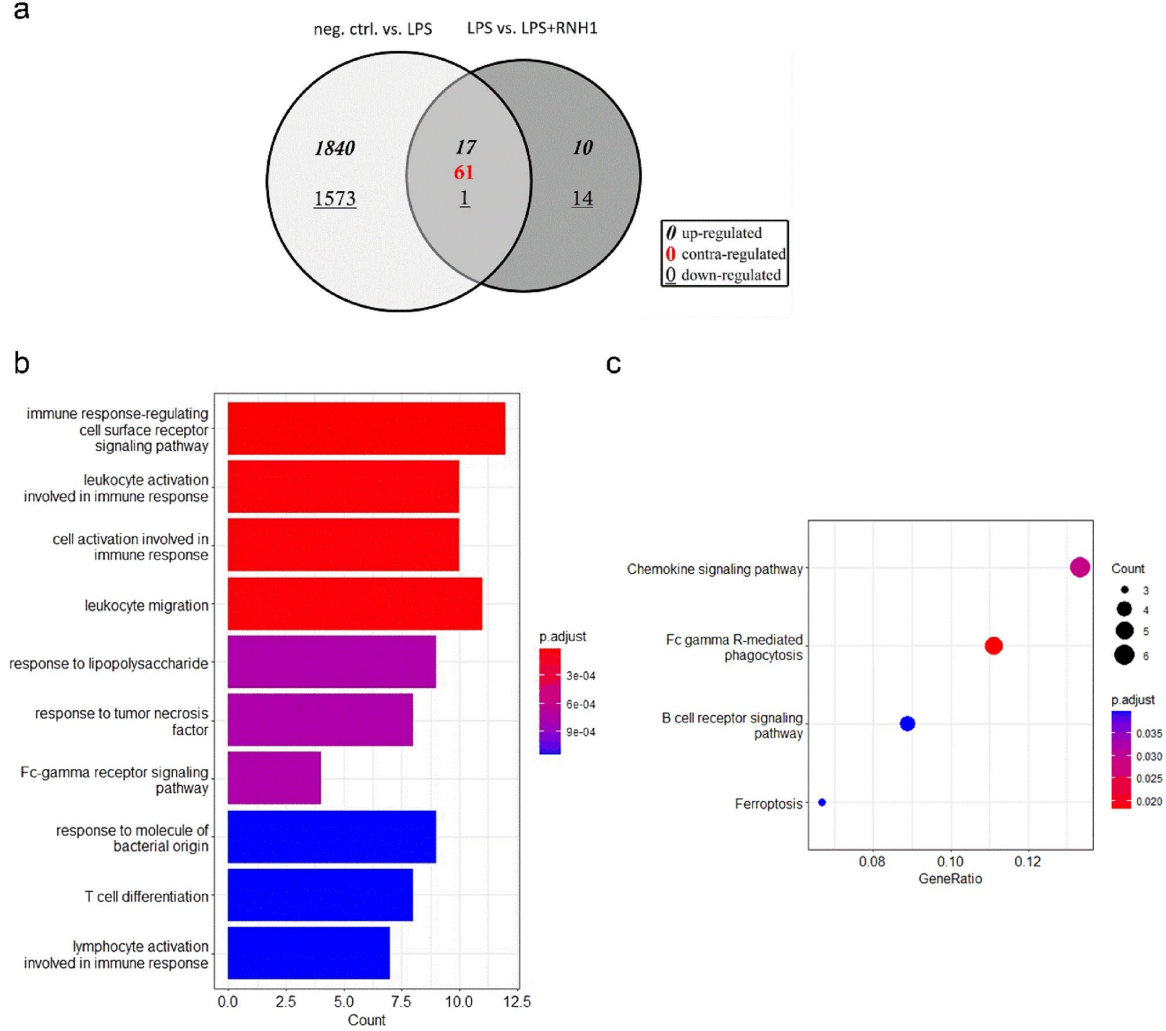
Identification and gene enrichment analysis of differentially expressed contra-regulated genes in PBMCs stimulated with 50 ng/ml LPS in the presence or absence of 640 U/ml RNH1 for 4 h compared to unstimulated cells. Presented are (**A**) a Venn diagram representing significantly differentially expressed genes in comparisons of neg. ctrl vs. LPS and LPS vs. LPS + RNH1; (**B**) Gene Ontology (GO) enrichment analysis according to the GO subclass “Biological Process”; and (**C**) KEGG pathway analysis of the 61 differentially expressed, contra-regulated genes. RNA sequencing was carried out in six biological replicates. RNH1, ribonuclease inhibitor 1; LPS, lipopolysaccharide; neg. ctrl., negative control; PBMCs, peripheral blood mononuclear cells.

After demonstrating an anti-inflammatory effect of RNH1, we aimed to gain further mechanistic insights utilizing next-generation sequencing. While 3,492 differentially expressed genes (DEGs) were identified between LPS-stimulated PBMCs and unstimulated cells (neg. ctrl.), the comparison of LPS and LPS + RNH1 treatment revealed 103 DEGs. Of these, 17 genes were upregulated, one was downregulated, and 61 genes were contra-regulated in both comparisons (Fig. 4a). Focused on contra-regulated DEGs, which are listed in Supplemental Table 3, our gene enrichment analysis revealed the top 10 Gene Ontology (GO) terms in the "Biological Process" subclass, primarily associated with the immune response, response to bacterial molecules, and Fc gamma receptor signaling (Fig. 4b).

Moreover, the matching of the contra-regulated DEGs to the Kyoto Encyclopedia of Genes and Genomes (KEGG) database revealed four significantly enriched pathways. While chemokine signaling showed the highest gene count and gene ratio, gene annotation to Fc gamma receptor-mediated phagocytosis was the most significant (p.adjust = 0.0156; Fig. 4c). Additionally, the pathways of B cell receptor signaling and ferroptosis were found to be enriched (Fig. 4c). The DEGs annotated in the respective pathways are shown in Supplemental Table 4.

### The effect of exogenous RNH1 on LPS-stimulated PBMCs regarding altered iron homeostasis/ferroptosis

**Figure 5:**
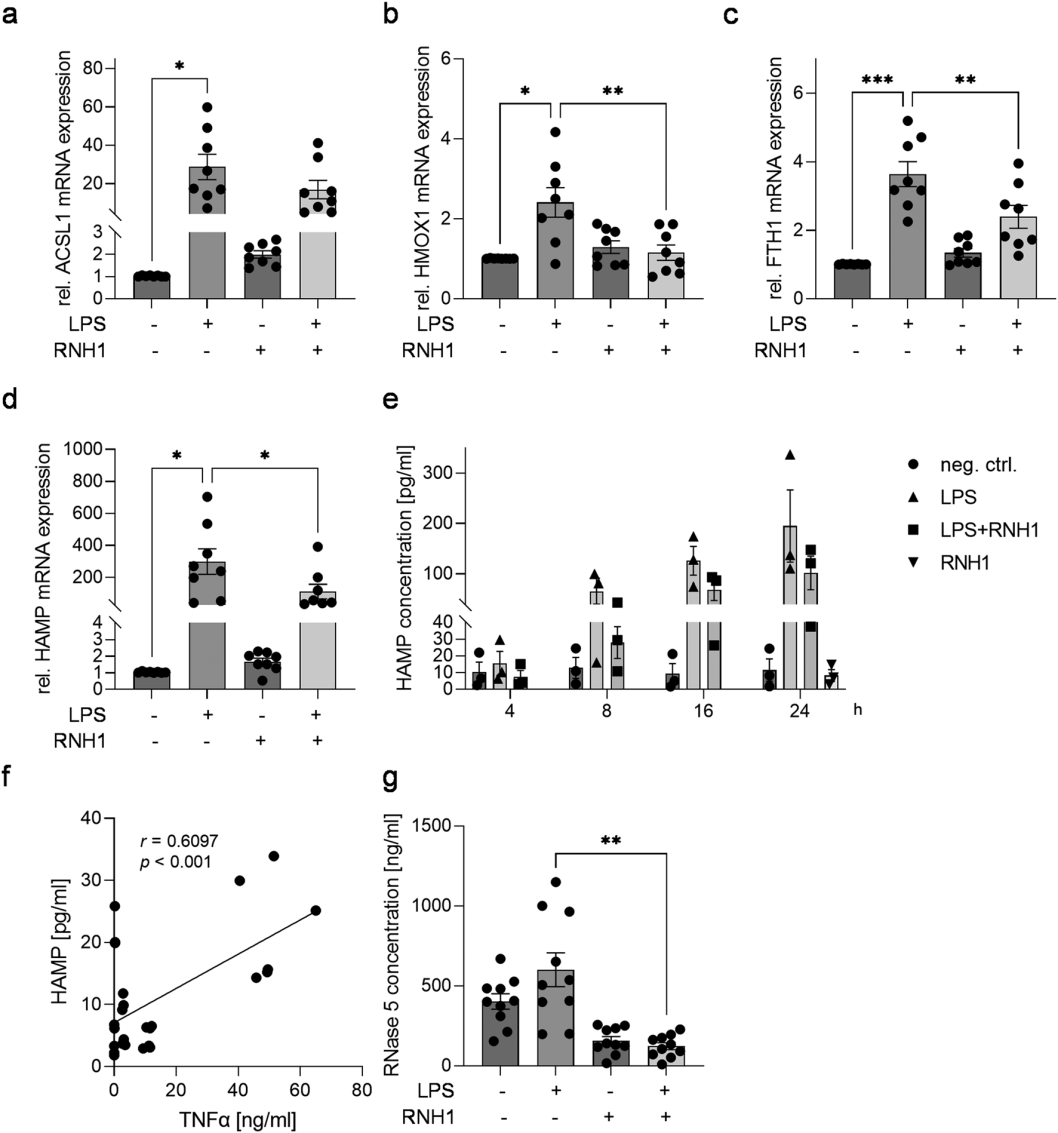
The effect of RNH1 on ferroptosis-associated genes in PBMCs challenged with LPS. Isolated PBMCs were exposed to 50 ng/ml LPS in the presence or absence of 640 U/ml RNH1. Unstimulated cells were used as neg. ctrl. Presented are (**A**) ACSL1, (**B**) HMOX1, (**C**) FTH1, and (**D**) HAMP relative mRNA expression levels measured using quantitative real-time PCR after stimulation for 4 h (n = 8); (**E**) HAMP protein concentration quantified using ELISA after 4, 8, 16, and 24 h (n = 3); (**F**) the correlation between TNFα and HAMP protein concentrations at 4 h (n = 3); and (**G**) RNase 5 protein concentration quantified using ELISA after 4 h (n = 10). Data are represented as column bar graphs of the mean ± SEM or XY dot plots with the regression line. Repeated-measures one-way ANOVA followed by Tukey’s test or simple linear regression were used for statistical analysis. *p < 0.05; **p < 0.01; ***p < 0.001; RNH1, ribonuclease inhibitor 1; LPS, lipopolysaccharide; PBMCs, peripheral blood mononuclear cells; ACSL1, long-chain acyl-CoA synthetase 1; HMOX1, heme oxygenase 1; FTH1, ferritin heavy chain 1; HAMP, hepcidin, RNase 5, ribonuclease 5.

Since ferroptosis and thus impaired iron homeostasis are associated with sepsis, we next aimed to verify the results of the pathway enrichment analysis by investigating the relative mRNA expression levels of the previously identified ferroptosis-associated gene cluster. The exposure of PBMCs to LPS for 4 h resulted in a significant increase in long-chain acyl-CoA synthetase 1 (ACSL1), HMOX1, and ferritin heavy chain 1 (FTH1) mRNA expression compared to unstimulated cells (*p* = 0.0165, *p* = 0.0281, and *p* = 0.0007; Fig. 5a–c). While stimulation with LPS + RNH1 significantly attenuated the relative mRNA expression of HMOX1 and FTH1 compared to LPS (*p* < 0.0023 and *p* = 0.0056, Fig. 5b,c), no significant alteration was found for ACSL1 (Fig. 5a). Additionally, we quantified the mRNA expression of HAMP, another contra-regulated DEG and a central iron-regulatory hormone (Supplemental Table 3). Analogous to HMOX1 and FTH1, LPS treatment significantly increased HAMP mRNA expression compared to untreated PBMCs (*p* = 0.0302). A significant decline in relative HAMP mRNA expression was observed after exposure to LPS + RNH1 compared to LPS (*p* = 0.0110; Fig. 5d).

HAMP levels are elevated in the serum of septic patients^32^. Thus, we investigated the role of RNH1 in sepsis-associated HAMP secretion. To this aim, PBMCs were challenged with LPS in the presence or absence of RNH1 for 4, 8, 16, and 24 h. LPS stimulation induced elevated HAMP secretion at all time points compared to unstimulated PBMCs. Thereby, LPS-induced HAMP levels increased in a time-dependent manner. Treating PBMCs with LPS + RNH1 reduced HAMP concentrations compared to LPS, irrespective of the stimulation duration (Fig. 5e). To investigate the relationship between inflammation and HAMP secretion, we analyzed the correlation of TNFα levels with HAMP levels in PBMC supernatants at 4 h. We found a significant correlation between TNFα and HAMP protein levels (*p* = 0.0007, r = 0.6097; Fig. 5f). Based on our clinical findings on the relationship between RNH1 and RNase 5, we also investigated RNase 5 levels. We measured higher RNase 5 concentrations in LPS-stimulated PBMCs compared to unstimulated cells. Significantly reduced RNase 5 protein concentrations were detected after stimulation with LPS + RNH1 compared to LPS (*p* = 0.0039; Fig. 5g).

### The effect of exogenous RNH1 on LPS-stimulated PBMCs regarding non-canonical inflammasome activation

**Figure 6:**
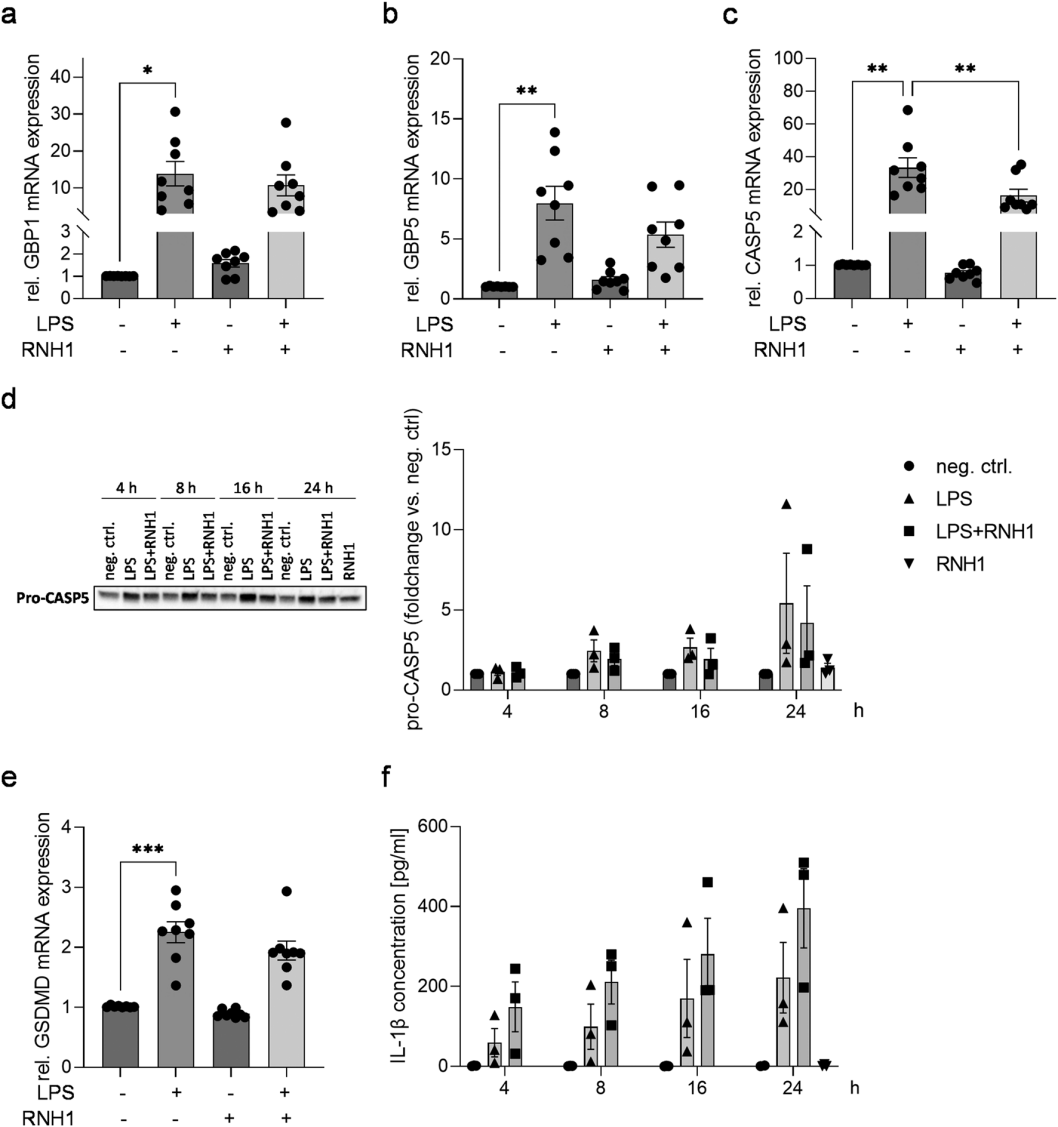
The effect of RNH1 on the regulation of LPS-induced relative mRNA expression of genes associated with non-canonical inflammasome activation and downstream signaling in PBMCs. Isolated PBMCs were exposed to 50 ng/ml LPS in the presence or absence of 640 U/ml RNH1. Unstimulated cells were used as neg. ctrl. Presented are (**A**) GBP1, (**B**) GBP5, and (**C**) CASP5 measured using quantitative real-time PCR (n = 8); (**D**) pro-CASP5 protein expression analyzed using Western blot after 4, 8, 16, and 24 h (n = 3); (**E**) relative GSDMD mRNA expression after 4 h (n = 8); and (**F**) IL-1β protein concentrations in PBMC supernatants quantified using ELISA after stimulation for 4, 8, 16, and 24 h (n = 3). Data are represented as column bar graphs of the mean ± SEM. Two-way ANOVA or repeated-measures ANOVA followed by Tukey’s test were used for multiple comparisons. *p < 0.05; **p < 0.01; RNH1, ribonuclease inhibitor 1; LPS, lipopolysaccharide; PBMCs, peripheral blood mononuclear cells; GBP1, guanylate-binding protein 1; GBP5, guanylate-binding protein 5; CASP5, caspase-5; GSDMD, gasdermin D; IL-1β, interleukin-1 β.

Recent evidence indicates that RNH1 inhibits inflammasome activation^33^. Among the 61 contra-regulated DEGs between neg. ctrl versus LPS and LPS versus LPS + RNH1, there was a gene cluster related to non-canonical inflammasome activation, including GBP1, GBP5, and CASP5 (Supplemental Table 3). To confirm the RNA sequencing results, we quantified the respective relative mRNA expression levels. LPS stimulation for 4 h resulted in a significantly elevated relative mRNA expression of GBP1, GBP5, and CASP5 compared to unstimulated PBMCs (*p* = 0.0237, *p* = 0.0069 and *p* = 0.0044, respectively; Fig. 6a–c). While the exposure to LPS + RNH1 significantly attenuated CASP5 mRNA expression compared to stimulation with LPS (*p* = 0.0027; Fig. 6c), relative GBP1 and GBP5 mRNA expression was not regulated (Fig. 6a,b).

After demonstrating a down-regulatory effect of RNH1 on LPS-induced relative CASP5 mRNA expression, we investigated its impact at the protein level. PBMCs challenged for 4, 8, 16, and 24 h showed increasing pro-CASP5 protein expression over time compared to unstimulated PBMCs. Regardless of the stimulation duration, LPS + RNH1 treatment attenuated pro-CASP5 protein expression compared to LPS (Fig. 6d).

Next, we explored RNH1’s impact on LPS-induced non-canonical inflammasome activation downstream of CASP5, assessing relative GSDMD mRNA expression and IL-1β secretion. After 4 h of LPS stimulation, relative GSDMD mRNA expression significantly increased compared to unstimulated cells (*p* = 0.0008). However, no alteration was observed after exposure to LPS + RNH1 compared to LPS (Fig. 6e). LPS stimulation increased IL-1β concentrations compared to unstimulated cells over time, with higher levels observed in longer LPS stimulation. Interestingly, exposure to LPS + RNH1 resulted in even higher IL-1β concentrations compared to LPS (Fig. 6f).

### Monocytes are the predominant type of immune cells that are LPS-positive

**Figure 7:**
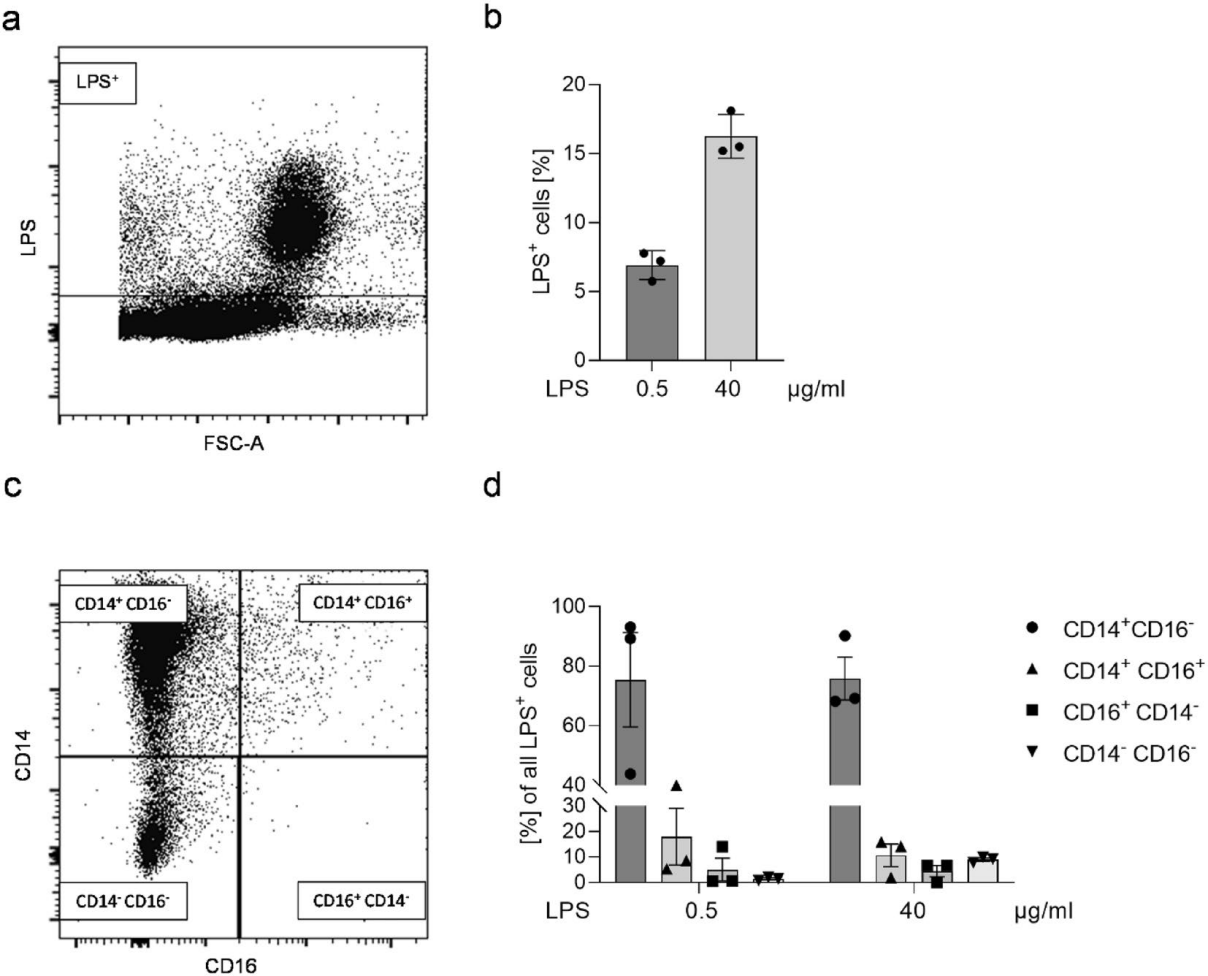
Identification of monocytes as the predominant type of immune cells that are LPS-positive. Isolated PBMCs were exposed to 0.5 µg or 40 µg/ml Alexa488-labeled LPS for 30 min. Presented are (**A**) a representative dot plot of LPS-positive immune cells after exposure to 40 µg/ml Alexa488-labeled LPS; (**B**) the proportion of LPS positive immune cells; (**C**) a representative dot plot of CD14^+^ CD16^−^, CD14^+^ CD16^+^ and CD14^−^ CD16^+^ monocyte subsets and CD14^−^ CD16^−^ cells from LPS-positive cells after exposure to 40 µg/ml Alexa488-labeled LPS; and (**D**) the proportion of CD14^+^ CD16^−^, CD14^+^ CD16^+^ and CD14^−^ CD16^+^ monocyte subsets and CD14^−^ CD16^−^ cells from LPS-positive cells. Data are represented as column bar graphs of the mean ± SEM (n = 3). LPS, lipopolysaccharide; PBMCs, peripheral blood mononuclear cells.

PBMCs contain different subsets of immune cells, mainly lymphocytes and monocytes. We investigated which cell type is mainly affected by LPS. All immune cells were included and analyzed regarding the proportion of LPS-positive immune cells after exposure to 0.5 or 40 µg/ml Alexa488-labeled LPS for 30 min. The gate setting for the immune cells is displayed in Supplemental Fig. 2a. Representative dot plots of LPS-positive immune cells are shown in Fig. 7a or Supplemental Fig. 2b. In fact, 6.92 ± 0.61% (low LPS dose) and 16.27 ± 0.92% (high LPS dose) LPS-positive cells were measured (Fig. 7b). Next, we analyzed the proportion of CD14^+^ CD16^−^, CD14^+^ CD16^+^ and CD14^−^ CD16^+^ monocyte subsets and CD14^−^ CD16^−^ cells from the LPS-positive cells. Representative dot plots of LPS-positive monocyte subsets and CD14^−^ CD16^−^ cells are shown in Fig. 7c or Supplemental Fig. 2c. We only detected 1.46 ± 0.37% (low LPS dose) and 8.99 ± 0.63% (high LPS dose) of non-monocytic cells (CD14^−^ CD16^−^) indicating that the majority of LPS-positive cells are monocytes (Fig. 7d).

### The effect of exogenous RNH1 on LPS-stimulated THP-1 macrophages regarding inflammation and non-canonical inflammasome activation

**Figure 8:**
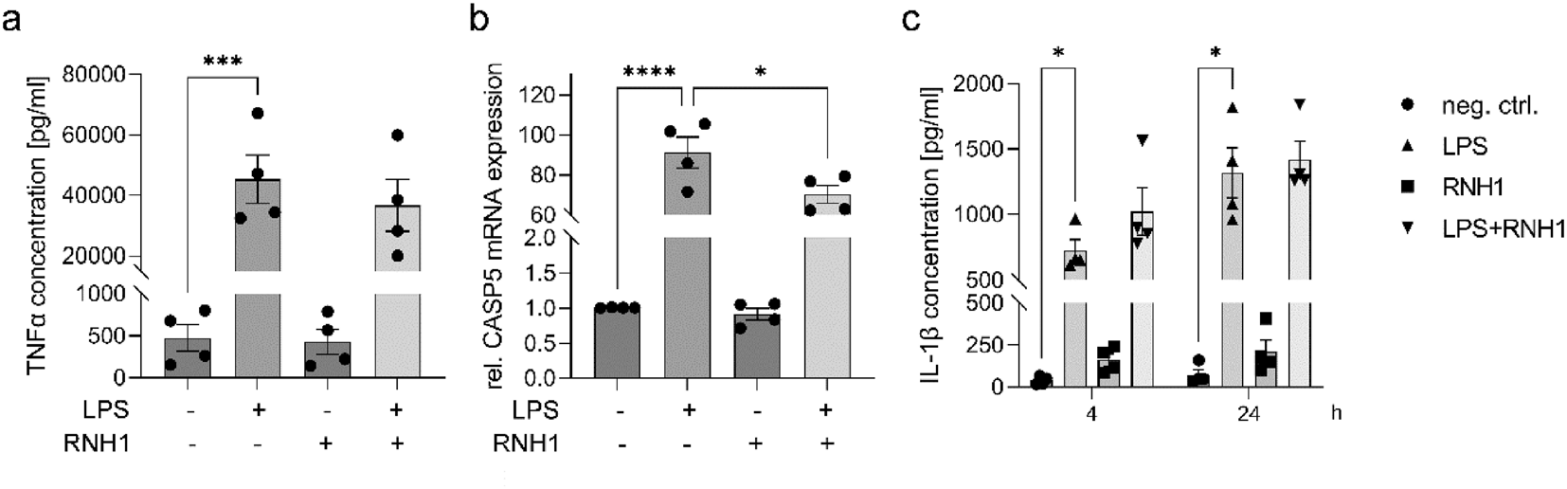
The effect of RNH1 on LPS-induced inflammation and non-canonical inflammasome activation in THP-1 macrophages. THP-1 macrophages exposed to 50 ng/ml LPS in the presence or absence of 640 U/ml RNH1 compared to unstimulated cells were analyzed regarding (**A**) TNFα protein concentrations after stimulation for 4 h determined using ELISA; (**B**) relative CASP5 mRNA expression after stimulation for 4 h quantified using ELISA; and (**C**) IL-1β protein concentration after stimulation for 4 and 24 h analyzed using ELISA. Data are represented as column bar graphs of the mean ± SEM. One-way ANOVA followed by Tukey’s test was used for multiple comparisons; *p < 0.05; ***p < 0.001; ****p < 0.0001; LPS, lipopolysaccharide; RNH1, ribonuclease inhibitor 1; TNFα, tumor necrosis factor α; CASP5, caspase-5; IL-1β, interleukin-1 β; neg. ctrl., negative control.

Since macrophages are crucial players during the immune response to sepsis and known to be involved in the LPS-induced non-canonical inflammasome response, we examined the role of RNH1 in LPS-induced inflammation and non-canonical inflammasome activation in THP-1 macrophages. Analogously to PBMCs, THP-1 macrophages were stimulated with LPS in the presence or absence of RNH1. In terms of inflammation, we measured significantly increased TNFα concentrations in THP-1 macrophages exposed to LPS for 4 h compared to unstimulated cells (*p* = 0.0008). However, stimulation with LPS + RNH1 had no significant effect on LPS-induced TNFα secretion compared to LPS (Fig. 8a). Analyzing non-canonical inflammasome activation, we observed a significant increase in relative CASP5 mRNA expression after exposure to LPS for 4 h compared to unstimulated THP-1 macrophages (*p* < 0.0001). Additional stimulation with LPS + RNH1 resulted in significantly decreased relative CASP5 mRNA expression levels compared to LPS-treated cells (*p* = 0.0292; Fig. 8b). Regarding IL-1β secretion, we measured a significant increase after LPS stimulation for 4 and 24 h compared to unstimulated cells (*p* = 0.0183 and *p* = 0.0309, respectively). At both time points, even higher IL-1β concentrations were detected in THP-1 macrophages after exposure to LPS + RNH1 compared to LPS (Fig. 8c).

## Discussion

Decades of interdisciplinary research have improved the understanding of sepsis pathomechanisms, yet evidence-based therapy lacks adjuvant treatment options in addition to causal and supportive interventions^34^. The recent focus on host response rather than pathogens led us to identify RNase 1 as a potential therapeutic target. Despite elevated eRNA and RNase 1 levels in septic patients, our research demonstrated that RNase 1 administration reduces cardiac apoptosis and septic cardiac dysfunction in vivo^21,23^. Interestingly, we also found significantly increased levels of RNH1, the inhibitor of RNase 1, and other ribonucleases in septic patients^23^. Since RNH1 is predominantly an intracellular protein for which no active release mechanism has yet been described in the literature, we hypothesize that RNH1 is passively released under certain conditions, such as cell injury or tissue damage. Generally, there is a paucity of knowledge regarding the physiological and pathophysiological role of RNH1 in sepsis.

Hence, we investigated RNH1 dynamics in patients on days 1–3, 5, and 7 after sepsis diagnosis. Compared with healthy subjects in whom RNH1 was not detectable, we measured elevated levels of RNH1 in the plasma of septic patients from day 1, aligning with the early pro-inflammatory phase. The persistently elevated RNH1 levels observed from day 3 onwards, despite no significant changes over time, could indicate an ongoing inflammatory response or a balance between pro- and anti-inflammatory reactions, which is characteristic of the later stages of sepsis. The peak in RNH1 levels observed on day 2, which correlates with increased mortality, supports a strong pro-inflammatory response. A systematic review and meta-analysis showed that the 30-day mortality in sepsis was 24.4%^35^. Within our patient cohort, 25% died, highlighting the representativeness of our study. In line with an earlier study demonstrating a correlation of RNH1 serum levels with in-hospital mortality in TAAA patients^28^, our results suggest that elevated RNH1 levels may be associated with worse outcomes in septic patients. The patient outcome is strongly dependent on whether the impairment or even failure of one or more organs occurs during the course of sepsis^3^. We examined RNH1's role in organ dysfunction in septic patients, finding a significant correlation between RNH1 levels and creatine kinase levels on day 3 when the SOFA score was highest. Among other, less serious conditions, creatine kinase is a marker of muscle necrosis and the release of intracellular muscle constituents into circulation, termed rhabdomyolysis^36^. During sepsis, rhabdomyolysis is caused by the direct invasion of the pathogen into the muscles and is associated with distinct forms of organ dysfunction, including cardiac dysrhythmia, liver failure, and AKI^37–39^. Consistent with our previous study demonstrating the good test accuracy of RNH1 for post-operative AKI in TAAA patients^28^, our results provide evidence that increased RNH1 levels are associated with sepsis-induced AKI. Regarding liver function, we observed significant correlations between RNH1 levels on day 3 and AST and ALT levels on day 5, aligning with hepatocellular damage often seen in late sepsis^40^. Consistent with this, we found that continued elevation of RNH1 correlated with higher bilirubin levels on day 7 and thus with worsening liver excretory function. This suggests elevated RNH1 as a potential early indicator of sepsis-associated liver dysfunction.

The significant correlations between RNH1 levels and markers of organ dysfunction at later time points, suggesting that the initial pro-inflammatory response has long-term impacts on organ function that manifest during the anti-inflammatory phase. While our findings initially reflect the pro-inflammatory phase of sepsis, they also highlight sustained inflammation or a complex interplay between pro- and anti-inflammatory processes during the course of the disease^41^. Future analyses, including cytokine responses such as IL-6, TNFα and IL-10 in relation to RNH1, could provide deeper insights into these dynamics.

Beyond sepsis, RNH1 has been identified as a potential biomarker in various cancer. For example, while higher levels of RNH1 have been associated with improved overall survival and recurrence-free survival in patients with lung adenocarcinoma^42^, elevated levels of RNH1 have been associated with shorter survival in patients with isocitrate dehydrogenase wild-type glioblastoma^43^.

RNase 5, known for its cytoprotective role post ER-stress-induced dissociation from RNH1 in kidney injury, showed no significant concentration differences over time or compared to healthy volunteers^44^. The lack of differences in the RNase 5 levels between patients and healthy volunteers was also described in connection with other diseases in a systematic review and meta-analysis from 2018. It was also clarified that this does not indicate that RNase 5 does not play a role in these diseases^45^. Interestingly, we found notable correlations between RNase 5 and RNH1 levels, but no correlations with RNase 1 or its activity. Therefore, elevated RNH1 in septic patients may result from host defense RNH1–RNase 5 dissociation to enable the cytoprotective function of RNase 5.

While past studies extensively explored RNH1’s role using knockout or overexpression^33,46,47^, our study uniquely employed RNH1 as a stimulus for the first time. This revealed a protective function, as RNH1 stimulation significantly reduced LPS-induced inflammatory response ex vivo. Building on previous findings of increased TNFα secretion in RNH1 knockout cells^33^, our results suggest exogenous RNH1 administration as a potential therapeutic approach for mitigating sepsis-associated inflammation.

Growing evidence links ferroptosis to sepsis and related organ damage. For instance, the ferroptotic marker glutathione peroxidase 4 was significantly decreased in the kidneys of mice with cecal ligation and puncture (CLP)-induced AKI^48^. It was recently reported that serum ferritin secretion in CLP mice required non-canonical inflammasome activation via CASP11, the murine homologue of CASP4/5^49^, thus underlining the tight connection between ferroptosis-associated genes and pyroptosis. Moreover, the absence of RNH1 after knockout was shown to increase NLRP3 inflammasome activation^33^. In line with this, we identified clusters of contra-regulated DEGs between the comparisons neg. ctrl. versus LPS and LPS versus LPS + RNH1 associated with ferroptosis and non-canonical inflammasome activation. Therefore, we suggested that stimulation with RNH1 may attenuate non-canonical inflammasome activation via the downregulation of CASP5 and subsequent ferroptosis via the regulation of FTH1 and other players involved in iron homeostasis.

Our results of the relative mRNA expression analysis successfully verified the LPS-mediated increase in CASP5, HMOX1, FTH1, and HAMP expression, as well as the diminishing effect of RNH1 on LPS-induced protein and/or mRNA expression. However, RNH1 treatment even increased LPS-induced inflammasome activation, as measured by IL-1β secretion, and had no regulatory effect on LPS-induced GSDMD expression. Notably, our experiments in diverse PBMCs, including T cells, B cells, natural killer cells, and monocytes, showed a small LPS-positive PBMC fraction, primarily affecting monocytes. Considering these findings and that prior studies on LPS sensing and non-canonical inflammasome activation focused on macrophages^50^, we investigated RNH1’s impact on LPS-induced CASP5 expression and IL-1β secretion in THP-1 macrophages and confirmed previous ex vivo results.

Contradictory views exist on the role of CASP5 in non-canonical inflammasome activation. Some argue that CASP4, not CASP5, is the functional homologue of murine CASP11, predominantly driving responses to intracellular LPS. However, other studies assert that CASP5 also plays a significant role in non-canonical inflammasome activation^10,51,52^. Our results indicating that the RNH1-mediated reduction of CASP5 is not sufficient to attenuate subsequent IL-1β release support the finding of Alexander-Floyd and colleagues that CASP4 and CASP5 can compensate for one another’s absence^51^. Consistently, we observed differential CASP4 expression in neg. ctrl. versus LPS, but not in LPS versus LPS + RNH1, indicating that RNH1 does not regulate LPS-induced CASP4 expression. However, it remains unclear whether the RNH1-mediated decrease in CASP5 is causally linked to the RNH1-mediated increase in LPS-induced IL-1β or contributes to other pathophysiological processes. Recent findings linked CASP4 and CASP5 to non-canonical inflammasome-induced extracellular vesicle (EV) secretion^53^.

Based on the RNH1-mediated increase in IL-1β secretion, which often links inflammation and ferroptosis^54^, we investigated whether treatment with RNH1 could amplify sepsis by interfering with the iron-regulatory host response. Transcription factor nuclear factor erythro2-related factor 2 (NFE2L2 or NRF2) is a crucial regulator of the innate immune response during sepsis^55,56^. Oxidative stress triggers NRF2 nuclear translocation, binding antioxidant response elements, and transcribing HMOX1, FTH1, and other genes^57^. The NRF2-mediated upregulation of HMOX1 facilitates the breakdown of heme, limiting the availability of free iron, while FTH1 sequesters excess iron within cells, collectively acting as a deterrent to ferroptotic processes. Since bilirubin is the result of heme degradation by HMOX-1^58^, it provides a link between our clinical observations and our ex vivo experiments, suggesting that RNH1 levels play a crucial role in iron homeostasis during sepsis. Interestingly, Hoang and colleagues reported that RNase 5 treatment activated the NRF2 pathway, protecting murine neurons from oxidative injury^59^. Notably, we identified NRF2 as a contra-regulated DEG between the comparisons neg. ctrl. versus LPS and LPS versus LPS + RNH1. Alongside FTH1 upregulation, elevated HAMP levels are a frequent pathophysiological observation in regard to altered iron homeostasis in septic patients^32^. In patients with septic shock, hepcidin was shown to be a predictor of 180-day mortality^20^. However, the administration of hepcidin reduced mortality and CLP-induced AKI in mice. Thus, the attenuating effect of RNH1 on LPS-induced relative FTH1 and HAMP mRNA expression and/or secretion may enhance rather than counteract sepsis. Our data provide strong evidence that exposure to exogenous RNH1 leads to the inhibition of RNase 5 released from LPS-induced dissociation from endogenous RNH1, hence preventing the anti-ferroptotic function of RNase 5 via NRF2–HMOX1–FTH1. This is further supported by the fact that the RNH1–RNase 5 complex is the strongest known binding of RNH1 with RNases^24^.

In line with studies linking FTH1 to reduced reactive oxygen species (ROS) formation and ROS-mediated HAMP downregulation^60,61^, we further suggest that the RNH1-induced reduction of FTH1 may lead to ROS accumulation, subsequently downregulating HAMP. Since RNase 5 is associated with reprogramming of macrophages to a proinflammatory phenotype, the inhibition of RNase 5 by RNH1 may also explain the RNH1-mediated reduction of TNFα release^62^. Proinflammatory cytokines such as interleukin-6 and TNFα exert regulatory effects on HAMP expression^63^, thus explaining the observed significant correlation between HAMP and TNFα concentrations. Moreover, it was reported earlier that treatment with RNase 5 significantly reduced the secretion of IL-1β in human macrophages^64^. Hence, the inhibition of RNase 5 by exogenous RNH1 may drive inflammasome activation, resulting in elevated IL-1β secretion.

In conclusion, our study demonstrates that (a) the plasma dynamics of RNH1 have significant potential as a biomarker for adverse outcomes in sepsis, including mortality and renal and hepatic dysfunction, underscoring its prognostic value in septic patients. (b) Furthermore, we explored correlations between RNase 5 and RNH1 levels, but no correlations with RNase 1 or its activity, revealing complex interactions that further improve our understanding of RNH1's role in sepsis. (c) Additionally, our ex vivo and in vitro experiments demonstrate that the treatment with RNH1 mitigated LPS-induced TNFα secretion and CASP5 expression, while increasing IL-1β secretion highlighting its contradictory modulatory effect on LPS-induced inflammation and non-canonical inflammasome activation and limiting its therapeutic use. However, exposure to exogenous RNH1 leads to the inhibition of RNase 5 released from LPS-induced dissociation from endogenous RNH1, hence preventing the anti-ferroptotic function of RNase 5 via NRF2–HMOX1–FTH1. The fact that bilirubin is the result of heme degradation by HMOX-1 provides a link between our clinical observations and our ex vivo experiments, suggesting that RNH1 level play a crucial role in iron homeostasis during sepsis. Therefore, our data contribute significantly to increasing the understanding of RNH1’s role in sepsis. Future research needs to further explore these relationships and the mechanistic pathways to potentially integrate RNH1 into clinical practice in the future and thus improve sepsis treatment.

## Limitations

The present study has several limitations. (a) In this study, we solely analyzed the effect of RNH1 on contra-regulated ferroptosis-associated genes and their cross-regulatory function with genes related to non-canonical inflammasome activation, neglecting the other pathways revealed in the KEGG gene enrichment analysis. Further studies need to investigate the role of RNH1 in chemokine signaling, Fc gamma receptor-mediated phagocytosis, and B cell receptor signaling under septic conditions. Moreover, further investigations should not be limited to contra-regulated DEGs. (b) Although we demonstrated the RNH1-mediated decrease in CASP5 expression, this study failed to demonstrate a causal association with any downstream effectors, like GSDMD or IL-1β. This demands further investigations addressing the role of the RNH1-mediated decrease in CASP5 in other pathophysiological processes, like EV secretion. (c) It should be noted that the mutual transferability between our clinical findings and the results of the ex vivo experiments is limited, as the average age of PBMC donors is considerably lower than that of sepsis patients. Further studies are needed to validate our results in age-matched experiments. (d) Despite our findings demonstrating the biomarker potential of RNH1 in sepsis-related renal and hepatic dysfunction, future research should explore the role of RNH1 in other organ dysfunctions, such as cardiac and pulmonary dysfunction, to provide a more comprehensive understanding of its role in sepsis-related multiple organ dysfunction syndrome.

## Methods

Detailed information on methods and materials can be found in the “Supplemental Data”.

### Ethics declarations

All samples were collected between 10/2020 and 11/2022 in a study carried out by the Department of Intensive Care and Intermediate Care at University Hospital RWTH Aachen after obtaining the informed consent of all study participants or their legal representatives and approval by the Ethics Committee of the University Hospital RWTH Aachen (EK100/20). This study was carried out in accordance with the Declaration of Helsinki.

### Study design

The blood samples of septic patients (n = 32) were collected on the day of (d1) and days 2, 3, 5, and 7 after diagnosis defined by sepsis-3. Individuals who were < 18 years old, were pregnant, or were receiving palliative care were excluded from the study.

### Isolation and stimulation of peripheral blood mononuclear cells

PBMCs from healthy blood donors (80% male, average age 38.25 years) were isolated from leukocyte reduction system chambers (EK 473/21). Due to blood donation regulations, the age limit for first-time donors is 60 years. For isolation, Ficoll density gradient centrifugation was utilized. Isolated PBMCs were cultured in Roswell Park Memorial Institute (RPMI) 1640 medium (ThermoFisher, Waltham, MA, USA) supplemented with 10% fetal bovine serum (FBS, Merck, Darmstadt, Germany) under a humified atmosphere of 5% CO_2_ at 37 °C. PBMCs were stimulated with 50 ng/ml LPS (Merck) in the presence or absence of 640 U/ml RNH1 (ThermoFisher). Unstimulated PBMCs served as a negative control.

### THP-1 cell culture and stimulation

THP-1 cells (ATCC, Wesel, Germany), a human leukemia monocytic cell line, were cultured in RPMI 1640 medium supplemented with 10% FBS, 1% penicillin/streptomycin (ThermoFisher), 1 mM sodium pyruvate (ThermoFisher), and 10 mM HEPES (ThermoFisher) under a humified atmosphere of 5% CO_2_ at 37 °C. All experiments were conducted in differentiated THP-1 cells, referred to as THP-1 macrophages, which were stimulated with 50 ng/ml LPS in the presence or absence of 640 U/ml RNH1 for 4 and 24 h. Unstimulated cells served as a negative control.

### Statistics

GraphPad Prism 9 (GraphPad Inc., San Diego, CA, USA) was used to perform the statistical analysis and to create the graphs. (Repeated) one-way or two-way ANOVA followed by Tukey’s test, an unpaired t-test (two-tailed), or simple linear regression were used for multiple comparisons with a significance level of *p* < 0.05. Data are presented as the mean ± SEM. The study population for clinical investigations was n = 32. In the ex vivo experiments, TNFα and RNase 5 ELISA assays were conducted with a sample size of n = 10. For the NGS experiments, the sample size was n = 6, while the qPCR experiments were carried out with n = 8 samples. Additionally, experiments involving IL-1 and HAMP ELISA, Western Blot, and flow cytometry were each conducted with a sample size of n = 3. All experiments using the THP1 cell line were performed with a sample size of n = 4.

### Supplementary Information

Supplementary Information 1.

Supplementary Information 2.

## Data Availability

All individual values for all data are available online in the Supporting Data file.
