# Supplementary material for: Ribonuclease inhibitor 1 emerges as a potential biomarker and modulates inflammation and iron homeostasis in sepsis
Source: Sci Rep. 2024 Jun 28;14:14972. doi: 10.1038/s41598-024-65778-8 (PMC11217267; doi:10.1038/s41598-024-65778-8)
Supplement: Supplementary file 1 — Supplementary Information 1. [file 41598_2024_65778_MOESM1_ESM.docx]

**Supplemental data**

**Ribonuclease inhibitor 1 emerges as a potential biomarker and modulates inflammation and iron homeostasis in sepsis**

Carolina Neu^1^, Christian Beckers^1^, Nadine Frank^1^, Katharina Thomas^1^, Matthias Bartneck^2^, Tim-Philipp Simon^1^, Jana Mossanen^1^, Kimmo Peters^1^, Tobias Singendonk^1^, Lukas Martin^1^, Gernot Marx^1^, Sandra Kraemer^1^ and Elisabeth Zechendorf^1*,†^

^1^ Department of Intensive and Intermediate Care, University Hospital RWTH Aachen, 52074 Aachen, Germany

^2^ Department of Medicine III, University Hospital RWTH Aachen, 52074 Aachen, Germany

^*^Correspondence: ezechendorf@ukaachen.de; University Hospital RWTH Aachen, Pauwelsstraße 30, 52074 Aachen, Germany, Tel.: +49-(0)-241-8035484, Fax: +49-(0)-241-8082056
^†^These authors contributed equally to this work.**Results**

**Supplemental Table 1** Patients’ characteristics

|  | **Healthy (n = 8)** | **Sepsis d1 (n = 32)** |
| --- | --- | --- |
| Age (year) IQR | 70 (61.25 – 83.25) | 63.50 (59.50 – 73.25) |
| Male sex (%) | 6 (75.00) | 21 (65.63) |
| Septic shock (%) | - | 20 (62.50) |
| Bacteria causing sepsis |  |  |
| Gram-negative | - | 6 (18.75) |
| Gram-positive | - | 12 (37.50) |
| Diabetes (%) | - | 14 (43.75) |
| Comorbidities (%) | - | 28 (87.50) |
| Reason of ICU admission (%) |  |  |
| Sepsis | - | 12 (37.50) |
| COVID-19 | - | 1 (3.13) |
| Surgery | - | 14 (43.75) |
| Cardiogenic disease | - | 9 (28.13) |
| Liver | - | 1 (3.13) |
| Polytrauma | - | 2 (6.25) |
| Tumor | - | 7 (21.88) |
| LOS (days) (IQR) | - | 34.00 (20.00 – 42.25) |
| LOS ICU (days) (IQR) | - | 16.00 (12.00 – 32.75) |
| ICU mortality (%) | - | 8 (25.00) |
| SOFA score (IQR) | - | 9.50 (7.25 – 12.00) |
| AKI (%) | - | 23 (71.88) |
| KDIGO stage (%) |  |  |
| 1 | - | 7 (21.88) |
| 2 | - | 1 (3.13) |
| 3 | - | 15 (46.88) |
| Creatinine (mg/dl) (IQR) | - | 1.35 (0.87 – 1.97) |
| Platelets (10^9^/l) (IQR) | - | 197.00 (123.5 – 342.0) |
| Leukocytes (10^9^/l) (IQR) | - | 14.00 (9.9 – 21.25) |
| CRP (mg/l) (IQR) | - | 178.00 (68.1 – 248) |
| PCT (ng/ml) (IQR) | - | 1.66 (0.51 – 9.00) |
| Lactate (mmol/l) (IQR) | - | 1.55 (1.1 – 2.08) |
| Creatine kinase (U/l) (IQR) | - | 202.00 (38.50 – 320.50) |

*Data are presented as n (%) or median (IQR). IQR = interquartile range (Q1 – Q3); LOS = length of stay; ICU = intensive care unit; SOFA =*[*Sepsis-Related Organ Failure Assessment; KDIGO = Kidney Disease: Improving Global Outcomes; CRP = C-reactive protein; PCT = procalcitonin*](https://www.bing.com/ck/a?!&&p=b6e13f9871833437JmltdHM9MTY5NDY0OTYwMCZpZ3VpZD0xZTdmYmU1OC03NjMxLTZkMGItMjZhMC1hYzg4Nzc1YTZjMzMmaW5zaWQ9NTYwMA&ptn=3&hsh=3&fclid=1e7fbe58-7631-6d0b-26a0-ac88775a6c33&psq=sofa+score&u=a1aHR0cHM6Ly9kZS53aWtpcGVkaWEub3JnL3dpa2kvU09GQS1TY29yZQ&ntb=1)

**Supplemental Table 2** Correlations between RNase 1, RNase 1 activity, RNase 5, and RNH1 in septic patients.

|  | **RNase 1 activity–RNase 1** | **RNase 1 activity–RNH1** | **RNH1–RNase 1** | **RNH1–RNase 5** |
| --- | --- | --- | --- | --- |
| d1 | **** p < 0.0001; r = 0.7793 | p > 0.05; r = 0.0637 | p > 0.05; r = 0.1454 | p > 0.05; r = 0.3386 |
| d2 | **** p < 0.0001; r = 0.7116 | p > 0.05; r = 0.0549 | p > 0.05; r = 0.1127 | p > 0.05; r = 0.2265 |
| d3 | **** p < 0.0001; r = 0.6839 | p > 0.05; r = 0.1524 | p > 0.05; r = 0.2629 | * p = 0.0133; r = 0.4623 |
| d5 | **** p < 0.0001; r = 0.8046 | p > 0.05; r = 0.0833 | p > 0.05; r = 0.2574 | p > 0.05; r = 0.3279 |
| d7 | **** p < 0.0001; r = 0.8248 | p > 0.05; r = -0,0815 | p > 0.05; r = 0.2411 | * p = 0.0398; r = 0.4413 |

*Simple linear regression was used for statistical analysis. RNase 1 = ribonuclease 1; RNase 5 = ribonuclease 5; RNH1 = ribonuclease inhibitor 1*


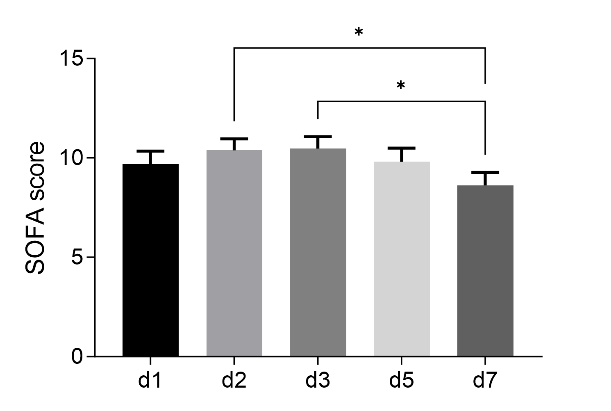


**Supplemental Fig. 1** Time course of the SOFA score in septic patients. Presented is the SOFA score of septic patients on day 1 – 3, 5 and 7 after diagnosis. Two-way ANOVA followed by Tukey’s test was used for statistical analysis. * p < 0.05; SOFA = sepsis-related organ failure assessment

| Supplemental Table 3 List of contra-regulated genes between the comparisons NK vs. LPS and LPS vs. LPS+RNH1 | | |
| --- | --- | --- |
| ENSEMBL ID | gene | gene name |
| ENSG00000116044 | NFE2L2 | nuclear factor erythroid 2-related factor 2 |
| ENSG00000143344 | RGL1 | ral guanine nucleotide dissociation stimulator like 1 |
| ENSG00000137331 | IER3 | immediate early response 3 |
| ENSG00000167703 | SLC43A2 | solute carrier family 43 member 2 |
| ENSG00000117228 | GBP1 | guanylate-binding protein 1 |
| ENSG00000131042 | LILRB2 | leukocyte immunoglobulin like receptor B2 |
| ENSG00000170017 | ALCAM | activated leukocyte cell adhesion molecule |
| ENSG00000196839 | ADA | adenosine deaminase |
| ENSG00000154451 | GBP5 | guanylate-binding protein 5 |
| [ENSG00000254087](https://www.ensembl.org/Homo_sapiens/geneview?gene=ENSG00000254087) | LYN | tyrosine-protein kinase Lyn |
| ENSG00000157227 | MMP14 | matrix metalloproteinase-14 |
| ENSG00000277443 | MARCKS | myristoylated alanine-rich C-kinase substrate |
| ENSG00000169715 | MT1E | metallothionein 1/2 |
| ENSG00000135678 | CPM | carboxypeptidase M |
| ENSG00000100647 | SUSD6 | sushi domain-containing protein 6 |
| ENSG00000114450 | GNB4 | guanine nucleotide-binding protein subunit beta-4 |
| ENSG00000182541 | LIMK2 | LIM domain kinase 2 |
| ENSG00000187116 | LILRA5 | leukocyte immunoglobulin-like receptor |
| ENSG00000003402 | CFLAR | CASP8 and FADD-like apoptosis regulator |
| ENSG00000133048 | CHI3L1 | chitinase-3-like protein 1/2 |
| ENSG00000136048 | DRAM1 | DNA damage-regulated autophagy modulator protein 1 |
| ENSG00000122224 | LY9 | lymphocyte antigen 9 |
| ENSG00000104972 | LILRB1 | leukocyte immunoglobulin like receptor B1 |
| ENSG00000101017 | CD40 | tumor necrosis factor receptor superfamily member 5 |
| ENSG00000189067 | LITAF | lipopolysaccharide-induced tumor necrosis factor-alpha factor |
| ENSG00000171049 | FPR2 | formyl peptide receptor-like |
| ENSG00000174837 | ADGRE1 | adhesion G protein-coupled receptor E1 |
| ENSG00000101336 | HCK | hemopoietic cell kinase |
| ENSG00000166523 | CLEC4E | C-type lectin domain family 4 member E |
| ENSG00000167996 | FTH1 | ferritin heavy chain |
| ENSG00000204103 | MAFB | transcription factor MAFB |
| ENSG00000132205 | EMILIN2 | elastin microfibril interfacer 2 |
| ENSG00000137757 | CASP5 | caspase 5 |
| ENSG00000161921 | CXCL16 | C-X-C motif chemokine 16 |
| ENSG00000151726 | ACSL1 | long-chain acyl-CoA synthetase |
| ENSG00000174238 | PITPNA | phosphatidylinositol transfer protein alpha |
| ENSG00000116741 | RGS2 | regulator of G-protein signaling 2 |
| ENSG00000152229 | PSTPIP2 | proline-serine-threonine phosphatase interacting protein 2 |
| ENSG00000166527 | CLEC4D | C-type lectin domain family 4 member D |
| ENSG00000171631 | P2RY6 | P2Y purinoceptor 6 |
| ENSG000001367167 | LCP1 | Lymphocyte cytosolic protein 1 |
| ENSG00000125753 | VASP | vasodilator-stimulated phosphoprotein |
| ENSG00000196912 | ANKRD36B | ankyrin repeat domain 36B |
| ENSG00000198829 | SUCNR1 | succinate receptor 1 |
| ENSG00000166920 | C15orf48 | chromosome 15 open reading frame 48 |
| ENSG00000171051 | FPR1 | formyl peptide receptor 1 |
| ENSG00000081059 | TCF7 | transcription factor 7 |
| ENSG00000105246 | EBI3 | interleukin 27 subunit beta |
| ENSG00000105697 | HAMP | hepcidin |
| ENSG00000123700 | KCNJ2 | potassium inwardly-rectifying channel subfamily J member 2 |
| ENSG00000274736 | CCL23 | C-C motif chemokine 15/23 |
| ENSG00000163220 | S100A9 | S100 calcium binding protein A9 |
| ENSG00000075624 | ACTB | actin beta |
| ENSG00000126262 | FFAR2 | free fatty acid receptor 2 |
| ENSG00000100453 | GZMB | granzyme B |
| ENSG00000107890 | ANKRD26 | ankyrin repeat domain-containing protein 26 |
| ENSG00000100292 | HMOX1 | heme oxygenase 1 |
| ENSG00000138755 | CXCL9 | C-X-C motif chemokine ligand 9 |
| ENSG00000112851 | ERBIN | erbb2-interacting protein |
| ENSG00000100697 | DICER1 | endoribonuclease Dicer |
| ENSG00000028277 | POU2F2 | POU class 2 homeobox 2B2:D63 |

| Supplemental Table 4 List of differentially expressed genes annotated to specific pathways according to KEGG enrichment analysis. | | | |
| --- | --- | --- | --- |
|  |  | differential gene expression (p.adjust) | |
| gene | pathway | NK vs. LPS | LPS vs. LPS+RNH1 |
| LYN | FC gamma R-mediated phagocytosis, chemokine signaling, B cell receptor signaling | ≤ 0.0001 | ≤ 0.001 |
| MARCKS | FC gamma R-mediated phagocytosis | ≤ 0.0001 | ≤ 0.05 |
| LIMK2 | FC gamma R-mediated phagocytosis | ≤ 0.0001 | ≤ 0.05 |
| HCK | FC gamma R-mediated phagocytosis, chemokine signaling | ≤ 0.0001 | ≤ 0.05 |
| VASP | FC gamma R-mediated phagocytosis | ≤ 0.0001 | ≤ 0.01 |
| GNB4 | Chemokine signaling | ≤ 0.0001 | ≤ 0.05 |
| CXCL16 | Chemokine signaling | ≤ 0.0001 | ≤ 0.05 |
| CCL23 | Chemokine signaling | ≤ 0.0001 | ≤ 0.05 |
| CXCL9 | Chemokine signaling | ≤ 0.01 | ≤ 0.05 |
| LILRB2 | B cell receptor signaling | ≤ 0.0001 | ≤ 0.05 |
| LILRA5 | B cell receptor signaling | ≤ 0.0001 | ≤ 0.0001 |
| LILRB1 | B cell receptor signaling | ≤ 0.0001 | ≤ 0.05 |
| HMOX1 | Ferroptosis | ≤ 0.01 | ≤ 0.0001 |
| FTH1 | Ferroptosis | ≤ 0.0001 | ≤ 0.05 |
| ACSL1 | Ferroptosis | ≤ 0.0001 | ≤ 0.01 |


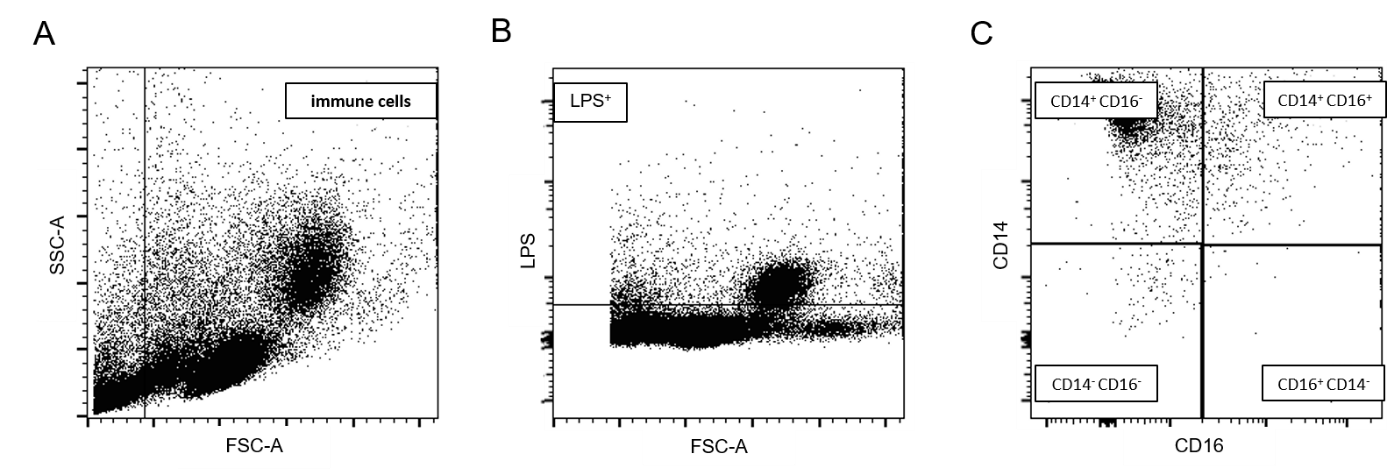


**Supplemental Fig. 2** Flow cytometric gate settings and LPS-positive monocytes after stimulation with 0.5 µg/ml Alexa488-labeled LPS. Presented are (A) the PBMC population containing lymphocytes and monocytes included for further analysis, (B) a representative dot plot of LPS-positive immune cells after exposure to 0.5 µg/ml Alexa488-labeled LPS and (C) a representative dot plot of CD14^+^ CD16^-^, CD14^+^ CD16^+^ and CD16^+^ CD14^-^ monocyte subsets and CD14^-^ CD16^-^ cells from LPS-positive cells after exposure to 0.5 µg/ml Alexa488-labeled LPS. LPS = lipopolysaccharide; PBMCs = peripheral blood mononuclear cells

**Methods**

**Study Design**

In this study, the blood samples of septic patients (n = 32) were collected on the day of (d1) and days 2, 3, 5, and 7 after diagnosis defined by sepsis-3. Individuals who were < 18 years old, were pregnant, or were receiving palliative care were excluded from the study. Patient characteristics, encompassing demographics, vital signs, laboratory values, blood gas analyses, and organ support, were retrieved from the patient data management system (Intellispace Critical Care and Anesthesia (ICCA) system, Philips, Amsterdam, Netherlands).

**Plasma Sampling**

Fifteen minutes after sample collection, EDTA-blood samples were centrifuged at 2000 g for 10 min and stored at −80 °C.

**Isolation and Stimulation of Peripheral Blood Mononuclear Cells**

PBMCs from healthy donors (80% male, average age 38.25 years) were isolated from leukocyte reduction system chambers (EK 473/21) utilizing Ficoll density gradient centrifugation. Isolated PBMCs were cultured in Roswell Park Memorial Institute 1640 (RPMI 1640) Medium (ThermoFisher, Waltham, MA, USA) supplemented with 10% fetal bovine serum (FBS, Merck, Darmstadt, Germany) under a humified atmosphere of 5% CO_2_ at 37 °C. PBMCs were stimulated with 50 ng/ml LPS (Merck) in the presence or absence of 640 U/ml RNH1 (ThermoFisher). Unstimulated PBMCs were used as a negative control.

**ELISA**

While a human RNase 1 ELISA kit (Sino Biological Inc., Beijing, China) was utilized to quantify RNase 1 plasma levels, the human RNase 5, TNFα, and IL-1β concentrations in cell supernatants were determined using DuoSet ELISA kits (Bio-Techne GmbH, Wiesbaden-Nordenstadt, Germany). All steps were performed according to the manufacturer’s instructions. As previously described, 96-well assay plates were coated with respective capture antibody diluted in PBS overnight. The plates were then blocked and incubated with the respective blocking solution for at least 1 h. Next, the standard and samples diluted in the respective dilution buffer were added to the plates for 2 h, followed by two-hour incubation with the respective detection antibody dilution. Assaying of RNase 5, TNFα, and IL1-β next required incubation with streptavidin–HRP for 20 min while protected from light. This step was not required for the assay used to measure RNase 1. After each step described, the plates were washed three times with a washing buffer (0.05% Tween® 20 (Merck) in PBS). Finally, the plates were incubated with either the Pierce^TM^ TMB substrate kit (ThermoFisher) for RNase 5, TNFα, and IL1-β assay or a substrate solution from the RNase 1 ELISA kit^1,2^. RNH1 plasma levels were quantified using the human RNH1 ELISA kit (MyBioSource, San Diego, CA, USA). All steps were performed according to the manufacturer’s instructions. In brief, pre-coated plates were washed two times before adding the standard and samples diluted in dilution buffer. After incubation for 90 min at 37 °C, the plate content was discarded and plates were incubated with a biotin-labeled detection antibody diluted in dilution buffer for 60 min at 37 °C, followed by three washing steps. Then, the streptavidin–HRP conjugate was added to the plates for 30 min at 37 °C. After five more washing steps, the plates were incubated with TMB substrate^3^. All reactions were terminated using 2 N H_2_SO_4_. Optical density was measured at 450 and 570 nm as reference wavelengths using a microplate reader (Infinity 200, Tecan, Männedorf, Switzerland).

**RNase 1 Activity Assay**

The RNase 1 activity in the plasma of septic patients was measured as previously described^4,5^. In brief, 100 µl plasma was mixed and incubated with 100 µl of 1 mg/ml poly (C), 47.5 µl RNase buffer (50 mM Tris pH 8, 130 mM NaCl, 2 mM EDTA, 0.1 mg/ml acetylated BSA), and 2.5 µl acetylated BSA for 15 min at 37 °C. Afterwards, 250 µl of ice-cold 6% perchloric acid containing 20 mM lanthanum-(III)-chloride and 100 µl of 10 mg/ml BSA were added to 100 µl reaction mixture and incubated on ice for 15 min. After centrifuging the reaction mixtures at 16,000 g for 15 min at 4 °C, poly (C) degradation in the supernatants was determined using absorbance measurement at 280 nm using a microplate reader (Infinity 200, Tecan). Samples were normalized to a standard curve of commercial RNase 1 (ThermoFisher) prepared according to the same assay procedure.

**RNA Sequencing and Differential Gene Expression Analysis**

Bcl2fastq (Illumina) was used for the generation of FASTQ files. Reproducible analysis was facilitated by sample processing using the publicly available nf-core/RNA-seq pipeline version 3.5 implemented in Nextflow 21.10.6 using Docker 20.10.12 with the minimal command^6-8^. Briefly, the trimming of lane-level reads was performed using Trim Galore 0.6.7 and they were aligned to the human genome (GRCh38.p13) using STAR 2.7.9a^9,10^. For gene-level and transcript-level quantification, Salmon v1.5.2 was utilized^11^. The previously described analyses were carried out utilizing custom scripts in R version 4.1.1, within the framework of DESeq2 v.1.32.0^12^. Contra-regulated DEGs were identified and visualized using the VennPlex software^13^. GO and KEGG gene enrichment analyses were performed using R version 4.3.1 and the clusterProfiler 4.8.2^14,15^, AnnotationDBI 1.62.2^16^, org.Hs.eg.db 3.17.0^17^ packages.

**Relative mRNA Expression**

As described previously, TRIzol reagent was used to isolate total RNA, followed by cDNA synthesis using a Maxima H Minus First Strand cDNA Synthesis kit (ThermoFisher). The mRNA expression was determined utilizing Power SYBR Green PCR Master Mix (Applied Biosystems, CA, USA) and the respective primers (Table 1) in a quantitative real-time PCR (StepOnePlus Real-Time PCR System, ThermoFisher). The mRNA expression levels were normalized against S7, and the relative mRNA expression was calculated using the 2-ΔΔCt method^2^.

**Supplemental Table 4.** Primers used for quantitative real-time PCR

|  | Forward primer | Reverse primer |
| --- | --- | --- |
| S7 | 5’-TCGTCTTTATCGCTCAGAGG-3’ | 5’-TCTCTTGCCCACAATTTCGC-3’ |
| ACSL1 | 5‘-CCATGAGCTGTTCCGGTATTT-3‘ | 5‘-CCGAAGCCCATAAGCGTGTT-3‘ |
| HMOX1 | 5‘-GGCCAGCAACAAAGTGCAAG-3‘ | 5‘-ATGGCATAAAGCCCTACAGCA-3‘ |
| FTH1 | 5’-AAGCTGCAGAACCAACGAGG-3’ | 5’-AGTCACACAAATGGGGGTCATT-3‘ |
| HAMP | 5‘-CAGCTGGATGCCCATGTTC-3‘ | 5‘-CAGCAGCCGCAGCAGAA-3‘ |
| GBP1 | 5‘-GTGGAACGTGTGAAAGCTGA-3‘ | 5‘-CAACTGGACCCTGTCGTTCT-3‘ |
| GBP5 | 5‘-AGGCCAAAGCAAGGTAGTGA-3‘ | 5‘-ATGATGCCACCTGGAAGAGT-3‘ |
| CASP5 | 5′-GGTGAAAAACATGGGGAACTC-3′ | 5′-TGAAGAACAGAAAGCAATGAAGT-3′ |
| GSDMD | 5′-GTGTGTCAACCTGTCTATCAAGG-3‘ | 5′-CATGGCATCGTAGAAGTGGAAG-3′ |

**Protein Isolation and Western Blot Analysis**

The Western blot analysis of CASP5 was performed in PBMC lysates. For protein isolation, PBMCs were washed twice with PBS. Cells were lysed in RIPA complete buffer (1x RIPA (Merck), 0.1% sodium dodecyl sulfate, 1x PIC (Roche, Mannheim, Germany), 1x PhosSTOP (Roche)) and incubated on ice for 30 min, followed by centrifugation at 11,000 g for 2 min at 4 °C. The protein concentrations in the supernatants were determined using detergent compatible protein assay (Bio-Rad, Hercules, CA, USA). Equal amounts of protein were separated by sodium dodecyl sulfate polyacrylamide gel electrophoresis. Then, a stain-free image was taken utilizing the ChemiDoc Imaging System (Bio-Rad) and separated proteins were transferred to a polyvinylidene difluoride membrane. The membrane was blocked in 5% milk in TBS containing 0.1% Tween® 20 (TBS-T) for at least 1 h. Next, the membrane was incubated with the CASP5 antibody (Cell Signaling, Danvers, MA, USA, 46680) diluted in 5% BSA in TBS-T over night at 4 °C. Five washing steps of 5 min each with TBS-T were performed, followed by incubation of the membrane with the HRP-conjugated secondary antibody for 1 h. After repeating the washing steps, protein bands were visualized using the SuperSignal ^TM^ West Femto Maximum Sensitivity Substrate (ThermoFisher) and ChemiDoc Imaging System (Bio-Rad). The densitometric analysis was performed utilizing the Image Lab software (Bio-Rad). The results were normalized against stain-free total protein amounts.

**Flow cytometry**

Isolated PBMCs were stimulated with 0.5 or 40 µg/ml Alexa488-labeled LPS (ThermoFisher) for 30 min shielded from light. Afterwards, cells were centrifuged at 400 g for 5 min, discarding supernatants, and resuspended in PBS. Another centrifugation step was followed by resuspending the cells in human FcR blocking reagent (Miltenyi, Bergisch Gladbach, Germany) diluted in FACS buffer containing 0.5 % NaN3 and 0.1 % BSA. Cells were incubated on ice for 10 min, before they were incubated with fluorochrome-conjugated antibodies to multicolor panels with CD14 and CD16 (both Miltenyi, 130-113-151 and 130-099-080) on ice for another 20 min. Next, cells were rinsed with FACS buffer twice. Cell suspensions were measured utilizing FACS Canto II flow cytometer (BD Bioscience, Heidelberg, Germany) and analyzed by FlowJo X software (TreeStar, Inc., Ashland, USA). First, cell populations were determined by including all immune cells by forward and side scatter. In the next step, LPS negative cells were excluded. LPS positive cells were further analyzed regarding their proportion of monocytes by subsequent analysis of CD14 and CD16.

**THP-1 cell culture and stimulation**

THP-1 cells (ATCC, Wesel, Germany), a human leukemia monocytic cell line, were cultured in RPMI 1640 supplemented with 10% FBS, 1% penicillin/streptomycin (ThermoFisher), 1 mM sodium pyruvate (ThermoFisher), and 10 mM HEPES (ThermoFisher) under a humified atmosphere of 5% CO_2_ at 37 °C. All experiments were conducted in differentiated THP-1 cells. The differentiation of THP-1 cells was performed by adding 100 ng/ml phorbol 12-myristate-13-acetate (PMA, Merck) for 48 h. After PMA exposure, THP-1 cells were cultured in PMA-free media for 24 h prior to stimulation. Differentiated THP-1 cells, referred to as THP-1 macrophages, were stimulated with 50 ng/ml LPS in the presence or absence of 640 U/ml RNH1 for 4 and 24 h. Unstimulated cells were used as a negative control.

**References**

1 Zechendorf, E. *et al.* A Potential Association between Ribonuclease 1 Dynamics in the Blood and the Outcome in COVID-19 Patients. *Int J Mol Sci* **24**, 10.3390/ijms241512428 (2023).

2 Neu, C. *et al.* DAMPs Released from Proinflammatory Macrophages Induce Inflammation in Cardiomyocytes via Activation of TLR4 and TNFR. *Int J Mol Sci* **23**, 10.3390/ijms232415522 (2022).

3 Zechendorf, E. *et al.* Ribonuclease 1 attenuates septic cardiomyopathy and cardiac apoptosis in a murine model of polymicrobial sepsis. *JCI Insight* **5**, 10.1172/jci.insight.131571 (2021).

4 Ohashi, A. *et al.* The expression and localization of RNase and RNase inhibitor in blood cells and vascular endothelial cells in homeostasis of the vascular system. *PLOS ONE* **12**, e0174237, 10.1371/journal.pone.0174237 (2017).

5 Reddi, K. K. & Holland, J. F. Elevated serum ribonuclease in patients with pancreatic cancer. *Proc Natl Acad Sci U S A* **73**, 2308-2310, 10.1073/pnas.73.7.2308 (1976).

6 Di Tommaso, P. *et al.* Nextflow enables reproducible computational workflows. *Nature Biotechnology* **35**, 316-319, 10.1038/nbt.3820 (2017).

7 Ewels, P. A. *et al.* The nf-core framework for community-curated bioinformatics pipelines. *Nat Biotechnol* **38**, 276-278, 10.1038/s41587-020-0439-x (2020).

8 Merkel, D. Docker: lightweight linux containers for consistent development and deployment. *Linux j* **239**, 2 (2014).

9 Dobin, A. *et al.* STAR: ultrafast universal RNA-seq aligner. *Bioinformatics* **29**, 15-21, 10.1093/bioinformatics/bts635 (2013).

10 Krueger, F., James, F. O., Ewels, P. A., Afyounian, E. & Schuster-Boeckler, B.

11 Patro, R., Duggal, G., Love, M. I., Irizarry, R. A. & Kingsford, C. Salmon provides fast and bias-aware quantification of transcript expression. *Nat Methods* **14**, 417-419, 10.1038/nmeth.4197 (2017).

12 Love, M. I., Huber, W. & Anders, S. Moderated estimation of fold change and dispersion for RNA-seq data with DESeq2. *Genome Biol* **15**, 550, 10.1186/s13059-014-0550-8 (2014).

13 Cai, H. *et al.* VennPlex–A Novel Venn Diagram Program for Comparing and Visualizing Datasets with Differentially Regulated Datapoints. *PLOS ONE* **8**, e53388, 10.1371/journal.pone.0053388 (2013).

14 Wu, T. *et al.* clusterProfiler 4.0: A universal enrichment tool for interpreting omics data. *The Innovation* **2**, 100141, <https://doi.org/10.1016/j.xinn.2021.100141> (2021).

15 Yu, G., Wang, L.-G., Han, Y. & He, Q.-Y. clusterProfiler: an R Package for Comparing Biological Themes Among Gene Clusters. *OMICS: A Journal of Integrative Biology* **16**, 284-287, 10.1089/omi.2011.0118 (2012).

16 Pages, H., Carlson, M., Falcon, S., Li, N. & Maintainer, M. B. P. Package ‘AnnotationDbi’. *Bioconductor Packag. Maint* (2017).

17 Carlson, M., Falcon, S., Pages, H. & Li, N. org. Hs. eg. db: Genome wide annotation for Human. *R package version* **3**, 3 (2019).
